# African Swine Fever Virus, Siberia, Russia, 2017

**DOI:** 10.3201/eid2404.171238

**Published:** 2018-04

**Authors:** Denis Kolbasov, Ilya Titov, Sodnom Tsybanov, Andrey Gogin, Alexander Malogolovkin

**Affiliations:** Federal Research Center for Virology and Microbiology, Pokrov, Russia

**Keywords:** African swine fever virus, viruses, genotype II, intergenic region variant, IGRI variant, African swine fever, swine, zoonoses, Irkutsk, Siberia, Russia

## Abstract

African swine fever (ASF) is arguably the most dangerous and emerging swine disease worldwide. ASF is a serious problem for the swine industry. The first case of ASF in Russia was reported in 2007. We report an outbreak of ASF in Siberia, Russia, in 2017.

African swine fever (ASF) is arguably the most dangerous swine disease worldwide. ASF virus (ASFV) is highly virulent for domestic swine and remains a global threat because no effective vaccine is available to eradicate the disease. The emergent potential of ASF has been demonstrated by its spread into Russia. In the 10 years since ASF was first diagnosed in the Caucasian region of Russia (*1*), the disease has reached Palearctic regions and is spreading into western Europe (*2*,*3*).

In 2017, the Federal Service for Veterinary and Phytosanitary Surveillance (Rosselkhoznadzor) reported, that during 2007−2017, >1,000 ASF outbreaks resulted in deaths of ≈800,000 pigs in 46 regions across Russia (*4*). Production of backyard swine industry decreased by almost half, from 1,119 tons of pork in 2007 to 608 tons of pork in 2017 (*5*). However, highly industrialized pig farms showed increased production every year during this same period, despite the ASF epidemic.

ASF has seriously affected and is actively spread by wild boar populations in Russia, but accurate numbers of boar killed by ASF or culling attempts are difficult to estimate. In June 2017, ASF was detected in the Czech Republic in 2 wild boar (*6*), demonstrating disease spread toward western Europe. In 2017, ASFV cases among backyard domestic pigs were detected in July in Romania (*7*), and later in October 2017 in Moldova (*8*). We report an outbreak of ASF in Far Eastern Russia.

Early in March 2017, an ASF outbreak was reported on 1 backyard farm in the Irkutsk region near the border with Mongolia (Figure) (*5*). All pigs had clinical signs typical of acute ASF, and 40 pigs died within 6 days of the appearance of the first clinical signs. In a 5-km risk zone established around the affected farm, 1,327 pigs were slaughtered within 3 days. Epidemiologic analysis showed that the farmer used table leftovers to feed pigs.

**Figure:**
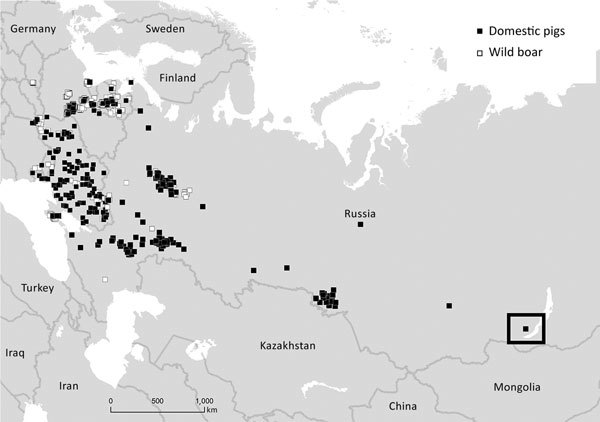
African swine fever outbreaks in Russia and countries in eastern Europe, 2017. Black box indicates outbreak in the Irkutsk region in Siberia, Russia.

ASFV DNA was identified by real-time PCR in the frozen pork products found on the farm. The origin of contaminated pork products is still under investigation. It is likely that ASFV-contaminated pork products provided a source of infection because these products are the most common source of ASF infection on backyard farms (*9*). ASF outbreaks nearest to the outbreak in Irkutsk occurred >4,000 km away in European Russia. Such a long geographic distance between ASF outbreaks within the country demonstrates that ASFV has a tremendous capacity for transboundary and transcontinental spread.

We identified the ASFV isolate from Irkutsk (ASFV/Irkutsk/dom/2017) by using nucleotide sequencing and molecular analysis. This isolate has capsid protein P72 genotype II and central variable region I and is an intergenic region (IGR) I variant (GenBank accession nos. KY963545, KY938010, and KY982843, respectively) according to the nomenclature of Gallardo et al. (*10*). The intergenic region between the *I73R* and *I329L* genes at the right end of the ASFV/Irkutsk/dom/2017 genome contains no additional tandem-repeat sequences. The ASFV IGRI variant is identical to the ASFV/Georgia/wb/2007 index isolate of the epidemic in Georgia in 2007 but represents an ASFV variant that is rare among recent ASFV isolates in Russia. In comparison, all recent ASF outbreaks in European Russia and eastern Europe have been caused by ASFV of the IGRII variant, which has an insertion of a tandem-repeat sequence in the intergenic region between the I173R and the I329L protein genes.

Our results indicate that an ASF outbreak in Siberia in 2017 was caused by the pan-Russian strain of ASFV (genotype II, central variable region I, and IGRI) that contains B646L (P72), B602L, and intergenic region I173−I329L sequences identical to those of ASFV index isolate ASFV/Georgia/wb/2007 (GenBank accession no. FR682468.1). ASFV-contaminated pork products still pose a major risk for transboundary emergence and spread of ASF. ASFV/Irkutsk/dom/2017 is a highly virulent strain and causes acute ASF in domestic swine. Since the outbreak in Irkutsk, subsequent ASF outbreaks have occurred in Siberia (March–October 2017) and near the border with China, raising concerns that ASF might be introduced into a population of 500 million pigs. This continued and far-reaching spread of ASF in Russia demonstrates the threat of disease emergence and increased spread worldwide.
